# Chronic viral coinfections differentially affect the likelihood of developing long COVID

**DOI:** 10.1172/JCI163669

**Published:** 2023-02-01

**Authors:** Michael J. Peluso, Tyler-Marie Deveau, Sadie E. Munter, Dylan Ryder, Amanda Buck, Gabriele Beck-Engeser, Fay Chan, Scott Lu, Sarah A. Goldberg, Rebecca Hoh, Viva Tai, Leonel Torres, Nikita S. Iyer, Monika Deswal, Lynn H. Ngo, Melissa Buitrago, Antonio Rodriguez, Jessica Y. Chen, Brandon C. Yee, Ahmed Chenna, John W. Winslow, Christos J. Petropoulos, Amelia N. Deitchman, Joanna Hellmuth, Matthew A. Spinelli, Matthew S. Durstenfeld, Priscilla Y. Hsue, J. Daniel Kelly, Jeffrey N. Martin, Steven G. Deeks, Peter W. Hunt, Timothy J. Henrich

**Affiliations:** 1 Division of HIV, Infectious Diseases, and Global Medicine,; 2 Division of Experimental Medicine, and; 3 Department of Epidemiology and Biostatistics, UCSF, San Francisco, California, USA.; 4 Monogram Biosciences Inc., South San Francisco, California, USA.; 5 School of Pharmacy,; 6 Department of Neurology, and; 7 Division of Cardiology, UCSF, San Francisco, California, USA.

**Keywords:** COVID-19, Adaptive immunity, Cytokines

## Abstract

**BACKGROUND:**

The presence and reactivation of chronic viral infections, such as EBV, CMV, and HIV, have been proposed as potential contributors to long COVID (LC), but studies in well-characterized postacute cohorts of individuals with COVID-19 over a longer time course consistent with current case definitions of LC are limited.

**METHODS:**

In a cohort of 280 adults with prior SARS-CoV-2 infection, we assessed the presence and types of LC symptoms and prior medical history (including COVID-19 history and HIV status) and performed serological testing for EBV and CMV using a commercial laboratory. We used covariate-adjusted binary logistic regression models to identify independent associations between variables and LC symptoms.

**RESULTS:**

We observed that LC symptoms, such as fatigue and neurocognitive dysfunction, at a median of 4 months following initial diagnosis were independently associated with serological evidence suggesting recent EBV reactivation (early antigen–diffuse IgG positivity) or high nuclear antigen (EBNA) IgG levels but not with ongoing EBV viremia. Serological evidence suggesting recent EBV reactivation (early antigen–diffuse IgG positivity) was most strongly associated with fatigue (OR = 2.12). Underlying HIV infection was also independently associated with neurocognitive LC (OR = 2.5). Interestingly, participants who had serologic evidence of prior CMV infection were less likely to develop neurocognitive LC (OR = 0.52).

**CONCLUSION:**

Overall, these findings suggest differential effects of chronic viral coinfections on the likelihood of developing LC and association with distinct syndromic patterns. Further assessment during the acute phase of COVID-19 is warranted.

**TRIAL REGISTRATION:**

Long-term Impact of Infection with Novel Coronavirus; ClinicalTrials.gov NCT04362150.

**FUNDING:**

This work was supported by NIH/National Institute of Allergy and Infectious Diseases grants (3R01AI141003-03S1, R01AI158013, and K24AI145806); the Zuckerberg San Francisco General Hospital Department of Medicine and Division of HIV, Infectious Diseases, and Global Medicine; and the UCSF-Bay Area Center for AIDS Research (P30-AI027763).

## Graphical abstract

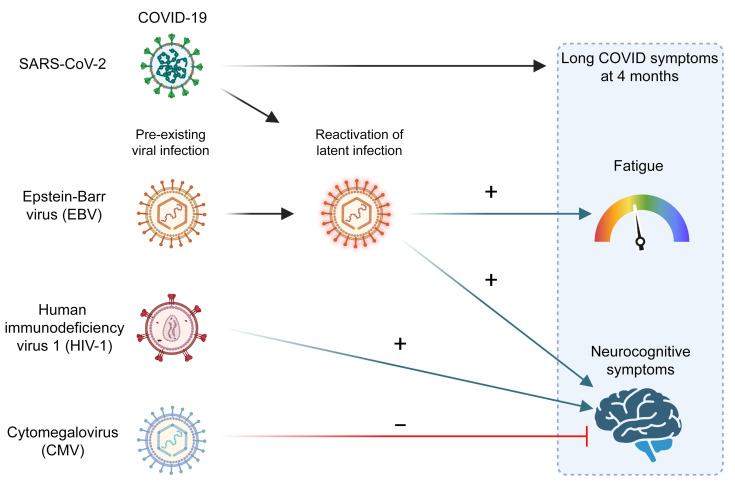

## Introduction

Intense efforts are underway to understand the pathophysiologic mechanisms that drive long COVID (LC), a type of postacute sequelae of SARS-CoV-2 infection (PASC), characterized by persistent or recurrent symptoms that interfere with quality of life (1, 2). Prior work has identified immune activation (3, 4), microvascular dysfunction (5, 6), autoimmunity (7, 8), and SARS-CoV-2 viral persistence (9–12) as mechanisms potentially contributing to LC. However, not all studies have confirmed these processes (13, 14), and identification of the determinants of PASC is essential to efforts to prevent and treat this condition (15).

Latent EBV is a ubiquitous human herpesvirus harbored by the vast majority (90%–95%) of adults in high-income settings; it is usually defined by the presence of detectable EBV viral capsid antigen (VCA) IgG levels (16). EBV can reactivate in immunocompromised individuals as well as in the setting of physiologic stressors, including acute infection (17). In some cases, EBV reactivated in tissues may not manifest with detectable circulating DNA in blood (18, 19). While reactivation of EBV is often considered to be a marker of physiologic stress rather than an independent pathophysiologic process, recent studies have demonstrated that EBV infection may drive multiple sclerosis (20), perhaps due to aberrant autoreactive immune responses to viral infection (21). Prior studies have demonstrated EBV reactivation, as defined by detectable circulating EBV DNA or EBV VCA IgM positivity, during acute SARS-CoV-2 infection (22–26). However, these studies typically involved hospitalized patients, and the high rates of reactivation (e.g., >80% of patients) were observed primarily in those receiving positive-pressure ventilation or other ICU-level care. Furthermore, VCA IgM levels wane rapidly and may not be useful outside the context of acute or subacute SARS-CoV-2 infection.

**Figure 1:**
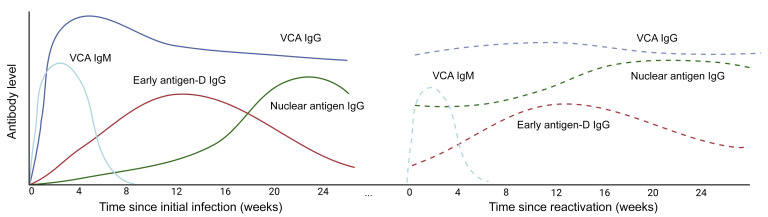
Schema of EBV-specific antibody responses during acute infection and hypothetical responses during SARS-CoV-2–related reactivation. EBV viral capsid antigen (VCA) IgM and IgG rise fairly early after acute infection, with VCA IgG levels persisting long-term. In contrast to VCA IgG levels, EBV nuclear antigen (EBNA) IgG levels rise more slowly following acute infection, at a time when virus changes from the lytic to latent phase of infection. Early antigen-D (EA-D) IgG responses rise early following acute infection but decay, often to low or undetectable levels, over many months. The dashed lines represent potential changes to antibody levels following EBV reactivation secondary to an insult, such as acute SARS-CoV-2 infection. EBV EA-D IgG responses and perhaps increases in baseline levels of EBNA IgG may be observed 3–4 months following reactivation.

EBV reactivation has also been proposed as a driver of LC. One small but highly provocative study identified EBV early antigen–diffuse (EA-D) IgG positivity among two-thirds of individuals experiencing LC (27). EBV EA-D IgG levels were higher in those with more LC symptoms, suggesting that recent EBV activity, as assessed with this measurement, might be associated with LC. Clinically, EBV EA-D IgG levels rise early after recent viral activity and generally remain positive for 3 to 6 months before decaying to undetectable levels in most (>85%) individuals (28, 29). In prior work, anti-EA antibodies, especially the diffuse component, have been considered to represent indirect evidence for lytic replication (30, 31). As a result, EBV EA-D IgG levels may act as a surrogate for recent EBV reactivation in tissues several months after the reactivation event (Figure 1). EBV also elicits life-long nuclear antigen (EBV nuclear antigen [EBNA]) IgG responses, which initially increase at the time of transition between the lytic and latent phases of acute EBV infection (28). Given a several-month lag in EBNA IgG responses following viral activity, it is possible that increases in EBNA IgG levels sampled months following COVID-19 onset in convalescent LC cohorts may act as a potential marker of EBV reactivation or other inflammatory insult at the time of acute SARS-CoV-2 infection. Furthermore, EBNAs can demonstrate molecular mimicry with host proteins and can elicit autoreactive immune responses (32–34). While hypothetical, it is possible that high-level EBNA IgG responses might represent a biologically important process related to EBV viral or immunologic activity, especially as autoimmune phenomena have been proposed as a potential mechanism of LC.

Recent work has shown that EBV DNA detectability during acute SARS-CoV-2 infection predicted the presence of symptoms at 30–60 days after COVID (7). Although limited by small sample size, sex imbalance, and overrepresentation of hospitalized individuals, as well as the relatively short duration of follow-up, these studies suggest that further investigation of the relationship between EBV-related pathology and LC is warranted. Studies controlling for potentially confounding factors in the interpretation of EBV reactivation and underlying chronic viral infections, such as timing of sample collection, hospitalization and severity of disease during initial infection, underlying health conditions, and other participant demographics, as well as studies accounting for the heterogeneity in syndromic patterns of LC that may reflect different disease phenotypes potentially caused by pathophysiologic mechanisms, are also needed.

Given the potential connection between EBV reactivation and the development of LC, there is also now much interest in how other underlying chronic viral infections, such as CMV and HIV, may influence both acute SARS-CoV-2 infection and postacute sequelae. For example, CMV seropositivity may be associated with more severe acute initial infection (35, 36), but it is not known whether CMV plays a significant role in LC. Recent data also demonstrated a potential link between the development of T cell receptor sequence repertoires and LC, suggesting that CMV cytolytic activity associated with gastrointestinal symptoms up to 2 months following acute infection (7), but direct evidence of CMV infection and LC are lacking. Similarly, we and others have recently observed that people with HIV may have a greater risk of developing LC (37, 38), but larger studies that control for factors such as human herpesvirus infections (many of which are enriched in people with HIV), participant demographics, and other underlying health conditions in both hospitalized and nonhospitalized participants are urgently needed.

In this study, we sought to investigate the prevalence of underlying CMV and HIV infection and serologic evidence suggesting EBV reactivation in a well-characterized postacute COVID-19 cohort of individuals with and without various LC symptoms (e.g., fatigue and neurocognitive, cardiopulmonary, and gastrointestinal symptoms) approximately 4 months following initial SARS-CoV-2 infection. We evaluated the independent associations among serologic evidence suggesting recent EBV reactivation, preexisting CMV infection, and a variety of different LC symptom groups controlled for clinical and demographic factors, including underlying HIV infection and details about acute infection. We hypothesized that the group experiencing LC symptoms would be enriched for evidence of EBV reactivation and underlying CMV seropositivity in comparison with individuals reporting complete recovery from COVID-19.

## Results

### Relationship between participant factors and LC symptoms.

Participant demographics, preexisting health conditions, COVID-19–related hospitalization, and EBV antibody test results were compared by LC symptom group in 280 participants at the time point beyond 60 days that was closest to 4 months (median, 123 days) following nucleic acid–based diagnosis of acute SARS-CoV-2 infection, with available data as shown in Table 1. Overall, the median age of participants was 45 years, 56% of participants were male at birth, 18% of participants had been hospitalized during acute infection, 65% of participants had a BMI of more than 30, and 19% of participants were living with HIV (the cohort was deliberately enriched for such individuals). In univariate analyses, there were significantly higher proportions of participants with LC or severe LC (reporting more than 5 symptoms) who had been hospitalized, compared with those without LC (21% and 26% versus 9%, respectively; all *P* < 0.05).

### Relationship between EBV serostatus and LC symptoms.

A higher proportion of participants who experienced LC or LC with more than 5 symptoms, compared with those without LC, had EBNA IgG levels greater than the quantitation limit of 600 U/mL (45% and 47% versus 28%; all *P* < 0.05).

**Figure 2:**
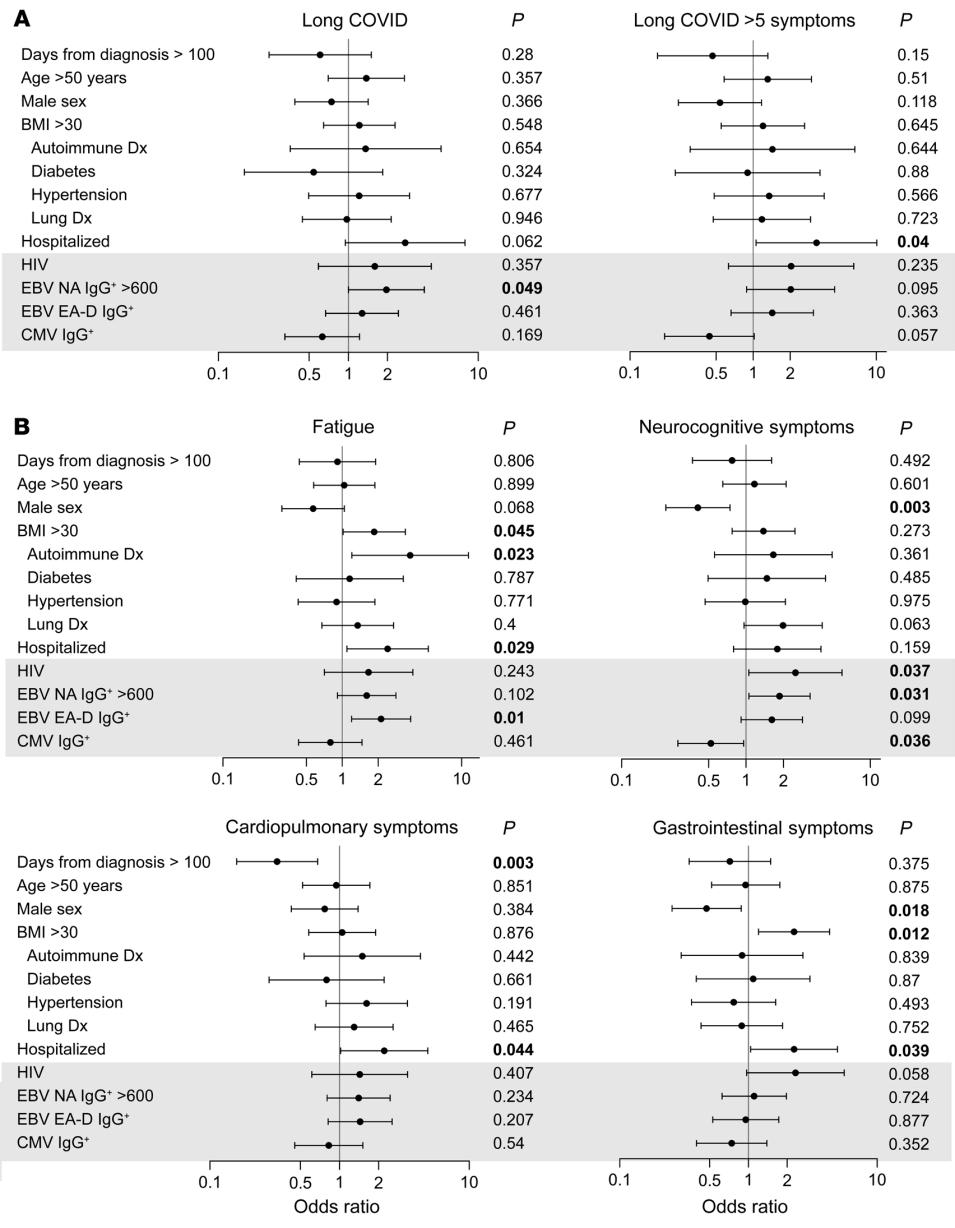
Results from covariate-adjusted logistic regression analysis of factors associated with long COVID and long COVID symptoms. Demographic characteristics, underlying health conditions, HIV and CMV positivity, and EBV serological results as predictors of participants with any persistent symptom (long COVID [LC]) or greater than 5 symptoms (LC > 5) across organ systems compared with those without LC are shown in **A**. Associations between covariates and fatigue, neurocognitive symptoms (sx), cardiopulmonary symptoms, and gastrointestinal symptoms are shown in **B**. *n* = 258 for all models with exception of LC > 5 symptoms (*n* = 153; participants with 1–4 LC symptoms were excluded from the analyses). Cases with missing values were excluded from the regression models. Dots and bars represent ORs and 95% confidence intervals. *P* values from regression analyses are shown adjusted for all covariates listed in the figure. VCA, viral capsid antigen; NA, nuclear antigen; EA-D, early antigen-D.

In order to determine the independent associations among demographic factors, preexisting medical conditions, and EBNA and EA-D IgG results with LC and in those with specific LC symptoms, we performed covariate-adjusted binary logistic regression modeling, as shown in Figure 2 (adjusted for timing of sample collection >100 days, prior COVID-related hospitalization, age >50 years, sex, BMI >30, preexisting diabetes mellitus, hypertension, renal disease, autoimmune disease, known HIV infection, CMV IgG seropositivity, EBNA IgG >600 U/mL, and EBV EA-D IgG positivity). Supplemental Figure 1 (supplemental material available online with this article; https://doi.org/10.1172/JCI163669DS1) shows a summary of the number of symptoms experienced by each participant in the various LC symptom groups, which was roughly similar overall across symptom groups (median range, 8–9.5, with significantly higher numbers of symptoms in those with gastrointestinal symptoms compared with those with neurocognitive symptoms). 258 participants (92%) with data available across all variables were included in logistic regression. EBV antibody variables were selected for inclusion in the final regression models based on antibody measures that may represent recent EBV reactivation, as recently reported (EBV EA-D IgG; ref. 27), or high levels of EBNA IgG (i.e., >600 U/mL, the upper limit of assay detection), based on the association with LC in univariate analysis (Table 1).

EBV VCA IgG positivity, VCA IgG above the limit of quantitation (>750 U/mL), and VCA IgM results were not significant across any analyses and were not included in the final models. Furthermore, very few participants had detectable VCA IgM levels (3.7%), which would be expected, as sampling was conducted months after acute infection.

In adjusted regression analyses, the odds of having LC with more than 5 symptoms, as well as LC characterized by fatigue, gastrointestinal symptoms, and cardiopulmonary symptoms, were higher in those who had been hospitalized during acute infection (Figure 2). Female sex also correlated with gastrointestinal and neurocognitive symptoms (Figure 2B).

Interestingly, participants reporting preexisting autoimmune disease (mainly thyroiditis) and those who had detectable EBV EA-D IgG responses had higher odds of experiencing fatigue (Figure 2B) a median of 4 months following COVID-19 diagnosis. Participants with high levels of EBNA IgG (>600 U/mL) had higher odds of experiencing neurocognitive symptoms. Furthermore, the EBNA IgG >600 U/mL ORs were higher in those with any number of LC symptoms (Figure 2A).

### EBV DNA measurements.

In order to determine if circulating EBV DNA is detectable during convalescence and whether any association between EBV DNA persistence and LC is present, we performed quantitative EBV PCR on plasma samples from a random subgroup of 50 participants who underwent EBV serological testing stratified by EA-D positivity (the subgroup demographics and participant phenotypes were similar to the larger cohort, as shown in Supplemental Table 1). Only 1 of the 50 participants had detectable plasma EBV DNA, and the level was below the limit of quantitation (<390 copies/mL). This participant had no reported preexisting medical conditions, had no detectable EA-D IgG or VCA IgM at the time of sampling, had EBNA and VCA IgG greater than the limits of quantitation, and reported 2 LC symptoms (persistent cough and heart palpitations) at the time of sampling.

### Relationship between CMV serostatus and LC symptoms.

Next, we analyzed the effect of CMV seropositivity on LC symptom clusters in the same covariate-adjusted regression models as above for EBV (Figure 2). CMV IgG positivity is not used to determine recent viral reactivation and is therefore solely a marker of preexisting CMV infection. In contrast to EBV serological results, after adjustment for potential confounders, CMV-seropositive participants had lower odds of developing neurocognitive LC (OR = 0.52, *P* = 0.036; Figure 2B). There was no evidence for an association between CMV serostatus and fatigue or any of the other nonneurologic LC symptom clusters.

### EBV and CMV serologies in participants with and without HIV.

Supplemental Table 2 shows the percentage of participants with and without HIV included in the regression analyses that had detectable EA-D IgG responses or EBNA IgG levels of more than 600 IU. Participants with HIV had a higher prevalence of EBV EA-D IgG antibodies compared with those without HIV (51.9% versus 32.1%, respectively, *P* < 0.01), but no significant difference was identified in EBNA IgG levels of more than 600 IU (44.2% versus 39.4%, respectively). As expected, nearly all participants with HIV had a positive CMV IgG (98.1%), as compared with 44.5% of those without HIV (*P* < 0.01 by 2-sided χ^2^ testing).

### Relationship between HIV and LC symptoms.

Of participants with HIV, 8 had viral loads above the limit of quantification (40 copies/mL; quantifiable values were 41, 50, 78, 424, 750, 28,118, 39,267, and 46,745 copies/mL). The median CD4^+^ T cell count and percentage were 576 (with a range of 404–785) and 32% (25%–38%), respectively. The median CD4/CD8 ratio was 0.86 (0.51–1.2). Twelve individuals had CD4^+^ T cell counts of below 350 cells/μL; of these, only 3 had CD4^+^ T cell counts of below 200 cells/μL. Participants with preexisting HIV had higher odds of neurocognitive (OR = 2.5, *P* = 0.037) and gastrointestinal symptoms (OR = 2.33, *P* = 0.058). We repeated covariate-adjusted analyses restricted to HIV-negative individuals (*n* = 213) as all but 1 participant with HIV (98.2%) was CMV IgG positive, compared with 54.8% of total participants included in the LC analyses. EBV EA-D IgG positivity remained significantly and positively associated with fatigue (OR = 2.26, *P* = 0.012), and CMV seropositivity remained significantly and negatively associated with neurocognitive PASC (OR = 0.51, *P* = 0.037).

### Analyses of nonhospitalized participants.

Many prior pathophysiological studies of postacute sequelae have included a majority of participants who were hospitalized for acute COVID-19, with many receiving intensive care and mechanical ventilation. There may also be a survival bias from including studies in which, those who develop LC, do so after severe initial disease presentations. As a result, we next performed regression analyses restricted to participants who did not require hospitalization (*n* = 211). Overall, the relationships observed in the total population between EBV and CMV serologies and other demographic factors and symptom clusters were similar. For example, the significant positive association between EBV EA-D IgG and fatigue strengthened (OR = 2.37, *P* = < 0.001). The negative associations between CMV and neurocognitive symptoms (OR = 0.53) and positive association between EBNA more than 600 U/mL (OR = 1.58) were similar to the larger cohort but lost statistical significance in the context of the smaller analysis population size.

### Association between EBV and CMV antibody results and circulating markers of inflammation.

**Figure 3:**
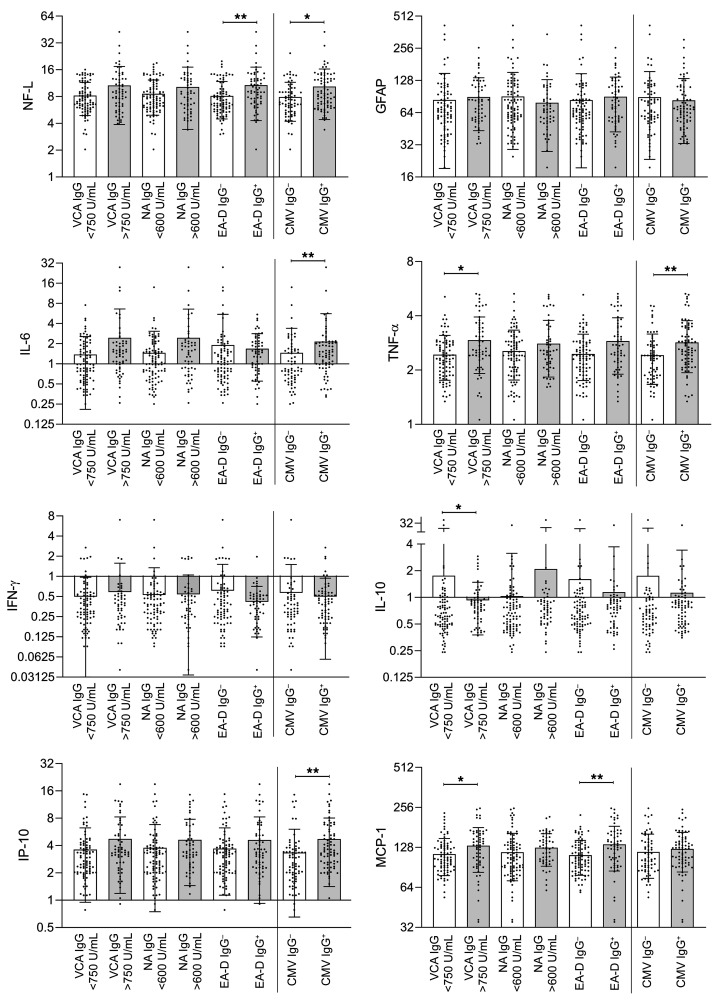
Circulating markers of inflammation grouped by EBV and CMV antibody result. Significant differences in levels of NF-L and MCP-1 were observed within each antibody group (e.g., EA-D IgG^+^ versus EA-D IgG^–^) by 2-sided Kruskal-Wallis testing with Dunn’s correction for multiple comparison (*P < 0.05, **P < 0.01). NF-L, IL-6, TNF-α, IP-10 were higher among those with CMV IgG^+^ compared to CMV IgG^–^. Bars and lines represent mean and standard deviation (all data points are shown). Units for plasma biomarkers are pg/mL. VCA, viral capsid antigen; NA, nuclear antigen; EA-D, early antigen-D.

We previously identified significant correlations between various markers of inflammation and LC symptoms, such as IL-6 and TNF-α (3, 39, 40). As a result, we examined the relationship between EBV and CMV antibody results in a subset of 143 participants (24 with HIV) who had available circulating biomarker data, as measured on the HD-X Simoa platform, including markers of neuronal injury, inflammation, and immune activation (glial fibrillary acidic protein [GFAP, a marker of astrocyte activation], neurofilament light chain [NFL, a marker of neuronal injury], monocyte chemoattractant protein-1 [MCP-1], IFN-γ, IL-6, IL-10, TNF-α, and IP-10). We identified significantly higher levels of NFL and MCP-1 in participants with measurable EBV EA-D IgG and significantly higher levels of TNF-α, IL-10, and MCP-1 in those with EBV VCA IgG levels of more than 750 U/mL in 2-sided, nonparametric analyses corrected for multiple comparisons (Figure 3). Despite having substantially lower odds of neurocognitive LC, CMV-seropositive participants had significantly higher plasma NFL, IL-6, IP-10, and TNF-α levels than those without CMV (Figure 3).

### Effect of CMV on associations between circulating markers of inflammation and LC symptoms.

Markers of inflammation, such as IL-6 and TNF-α, have been previously associated with LC and PASC and were elevated in participants with underlying CMV infection, as above (3, 4, 7). However, CMV was negatively associated with LC outcomes in our regression modeling, and to help clarify the relationships between biomarkers and CMV as predictors of LC, we performed binary logistic regression, including each biomarker alone or covariate adjusted with CMV IgG positivity with LC symptom clusters, as shown in Supplemental Table 3 (*n* = 141 with all data available). Interestingly, adjusting for CMV status strengthened the associations between inflammation and LC.

## Discussion

In a cohort of several hundred individuals with confirmed prior SARS-CoV-2 infection, we found that certain factors associated with chronic viral infections, such as serologic evidence suggesting recent EBV reactivation and preexisting HIV infection, were independently associated with higher odds of various LC symptom clusters. In contrast, participants who had serologic evidence of prior CMV infection were less likely to report neurocognitive symptoms and tended to have less LC overall. Of note, we identified LC even in those without evidence of EBV reactivation or CMV positivity, suggesting that these factors are not essential to the development of persistent symptoms or sequelae.

Our study confirms and extends prior studies that identified an association between EBV EA-D positivity and LC symptoms, raising the intriguing hypothesis that EBV reactivation may be mechanistically related to specific LC syndromic phenotypes. By carefully defining such phenotypes and adjusting for various participant factors, sample timing, underlying health conditions, and prior hospitalization, we identified a strong association between evidence suggesting recent EBV reactivation and fatigue, one of the most prevalent LC symptoms. We were able to demonstrate that serologic evidence suggesting recent EBV reactivation may be specifically associated with fatigue and neurologic symptoms but less so with other LC syndromic phenotypes (i.e., cardiopulmonary and gastrointestinal symptoms). In analyses excluding participants who were hospitalized, we were able to confirm that these associations were not entirely due to differences in acute COVID-19 severity. Whether EBV reactivation was the root cause of these symptoms, it should be noted that primary EBV infection (e.g., mononucleosis) may lead to prolonged fatigue, and EBV seroconversion has recently been shown to be common prior to the development of multiple sclerosis, an autoimmune condition that may be precipitated by aberrant, autoreactive immune responses to this virus (20). Because autoimmunity has been proposed as a pathophysiologic mechanism underlying LC (7, 15) and preexisting autoimmunity was associated with LC in our analysis, further study of its potential relationship with EBV disease activity in this patient population is warranted.

The biological mechanisms leading to high levels of EBNA IgG (greater than the assay limit of detection of 600 U/mL) observed in association with LC symptoms and neurocognitive symptoms are not entirely clear. Whereas EA-D IgG responses are generally understood to be a result of recent EBV reactivation in those with preexisting latent EBV infection (27), nearly 90% of our cohort had detectable EBNA IgG, consistent with the long-lasting nature of this antibody and high proportion of participants with preexisting EBV infection. It is possible that those with higher levels experienced a recent increase following EBV reactivation, but given the lack of sampling during or before acute SARS-CoV-2 infection, we do not know for certain. Nonetheless, EBNA IgG responses usually peak during establishment (or perhaps reestablishment) of EBV latency (16, 17, 28), the timing of which is consistent with the postacute sample collection timing here. The EBNA IgG assay used in this study is specific for EBNA1, which plays an important role in facilitating latent EBV infection (41). EBNA1 and EBNA2 expressed during either the latent or lytic phase of EBV infection has known molecular mimicry with host proteins that may lead to autoantibodies that have been implicated in various diseases, such as multiple sclerosis (32, 42). For example, cross reactivity between EBNA1 and glial cell adhesion molecules has recently been reported in the setting of multiple sclerosis (34). EBV antibodies have also long been associated with myalgic encephalomyelitis/chronic fatigue syndrome, although a definitive causal link is lacking (43). It is also possible that high EBNA IgG levels resulted from nonspecific hypergammaglobulinemia that can develop during acute viral infections or from higher tissue or circulating latently EBV-infected memory B lymphocyte burden, a cellular compartment expanded in COVID-19 (44). Whereas EBV reactivation in the throat has been shown to be associated with fatigue (45), further studies involving tissue-derived or circulating mononuclear cells in convalescent cohorts following acute infection to determine viral cell burden and EBV/CMV-specific T cell responses are needed.

A recent study (46) showed that, in addition to EBV EA-D antibody responses, seropositivity of EBV envelope glycoproteins gp42 and gp350, which are essential for EBV lytic infection of B cells, were enriched in participants with LC (46). These EBV envelope proteins are targets of neutralizing antibodies (47) and, in the case of gp350, may be short-lived in circulation, suggesting recent viral activity prior to or following the development of LC.

We made the surprising and potentially novel observation that CMV seropositivity was negatively associated with the development of LC phenotypes. The mechanism underlying this observation is not immediately clear, and we can only speculate on possible explanations. It is plausible that CMV-seropositive individuals might mount more robust adaptive immune responses to SARS-CoV-2. For example, CMV seropositivity in younger adults is actually associated with heightened adaptive immune responses to influenza vaccination (48), despite earlier studies in the aging literature linking CMV to immunosenescence phenotypes (49). Alternatively, CMV-induced immunoregulatory pathways, including secretion of its own viral IL-10, might dampen local inflammation in areas of CMV reactivation, decreasing the risk of autoantibody formation (to the extent that autoantibodies may contribute to the risk of neurologic LC symptoms) (50, 51). It is also unclear whether these associations reflect a direct causal effect of CMV on LC risk or host factors that affect the risk of CMV infection and LC independently. It is interesting that CMV serostatus was more strongly associated with neurologic LC symptoms than other syndromic phenotypes. While CMV-infected myeloid cells can be found in the central nervous system and CMV-induced inflammation might plausibly affect blood-brain barrier permeability (52), it is not immediately clear why CMV status would be so specifically linked to neurologic as opposed to nonneurologic LC symptoms. Finally, why two chronic herpesvirus infections — EBV and CMV — have qualitatively different associations with LC remains entirely unclear, though perhaps the anatomic localization of herpesvirus reactivation is an important factor. For example, EBV preferentially reactivates within B cell follicles, where antibody responses develop, while CMV preferentially reactivates elsewhere (53).

It is particularly interesting that CMV seropositivity is associated with decreased odds of developing LC but worse disease severity in acute COVID-19, as reported in some recent studies (35, 36). Although CMV seropositivity was not completely protective against LC in our study, the differential effects of CMV serostatus on acute COVID versus LC suggest that assessment of CMV serostatus may be important in future mechanistic evaluations of COVID-19. Indeed, CMV seropositivity is associated with increased systemic inflammation, but a decreased odds of LC (3, 39). This finding suggests that sources of inflammation unrelated to CMV may be driving LC risk in COVID-19 survivors and highlights the importance of the source of inflammation — as opposed to simply systemic inflammation itself — in mediating the risk of LC.

It is also notable that HIV infection was independently associated with the development of neurologic LC and, to a lesser degree, gastrointestinal symptoms but not other LC syndromic phenotypes (e.g., fatigue, which was more closely linked to serologic evidence suggesting recent EBV reactivation). We and others have previously demonstrated that markers of persistent immune activation and inflammation are elevated in the setting of LC (3, 4, 7, 39, 46). Interestingly, chronic HIV-1 infection, even in the setting of long-term suppressive antiretroviral therapy, leads to increases in similar inflammation markers such as IL-6 and C-reactive protein (54). As a result, preexisting HIV infection and the baseline elevations in inflammation and aberrant immune responses may predispose people to developing LC (55). Furthermore, EBV EA-D IgG positivity was significantly higher in participants with HIV, a condition which could have led to more robust EBV reactivation and subsequent LC symptoms.

Thus, each chronic viral infection assessed in our study not only affected the odds of LC, but also exhibited specific and distinct syndromic associations. Whichever mechanisms explain these findings, these observations highlight the importance of measuring specific LC syndromic phenotypes, as their underlying pathogenic mechanisms may well be distinct. They also highlight the likely heterogeneous nature of LC and may help determine inclusion in various future interventional trials. In fact, it will likely be difficult to prove any causal or modifying role of LC (e.g., EBV reactivation, CMV serostatus, long-term SARS-CoV-2 viral persistence, autoreactive immunity, etc.) without measuring the effects of targeted interventions in well-designed studies. Furthermore, given that there is paucity of circulating EBV during convalescence, the potential effect of EBV reactivation on the development of LC is likely to be greatest during acute COVID-19, and factors such as this will need to be considered in the design of such interventional studies.

Strengths of this study include the large sample of well-characterized patients with postacute COVID-19, most of whom were not hospitalized during acute infection, at a time point consistent with consensus case definitions of LC. Nevertheless, this study has several limitations. Although diverse, our cohort is a convenience sample not representative of all individuals with COVID-19 or LC. In particular, while we specifically oversampled people with treated HIV infection to assess its association with LC, we have a limited subsample of people with HIV to detect modest effect sizes. We also did not have access to biospecimens from acute or very early convalescent infection (<30 days), which is the time period in which latent EBV infection is most likely to reactivate. Because of this, we relied upon the use of serological evidence suggesting recent EBV reactivation, which remains hypothetical. For example, while EA-D IgG generally becomes undetectable after 6 months in most individuals, up to 20% of healthy people may have these antibodies for years (29). These serological measures are imprecise and do not provide a clear time course of EBV reactivation events. As a result, direct evaluation of EBV dynamics during the early phase of SARS-CoV-2 infection is warranted, although we believe our results demonstrating limited viremia during the postacute stage strongly suggest that investigation of EBV viremia during this time period is of limited utility. In addition, because of challenges in collecting samples during acute COVID-19, further work defining the dynamics of EBV serological profiles suggestive of recent reactivation would be of benefit to the field. We also note that this study was exploratory in nature, with multiple statistical comparisons conducted, and that the potential for a type 1 error is high. We performed statistical adjustments whenever possible. Although a type 1 error could be present, the differential associations between the 3 chronic viral infections and the various syndromic phenotypes suggest more specificity to the biologic associations than just random noise. Finally, EBV and CMV reactivation are often tissue-based processes, and such samples may be needed in order to identify persistent, smoldering infection. As a result, tissue studies will be critical to understanding the full pathophysiological mechanisms underlying LC.

In summary, this study expands our understanding of the relationships between chronic viral infections and the odds of distinct LC syndromic phenotypes. While it remains unclear whether these associations reflect causal effects of viral coinfections or host factors associated with viral coinfections on LC, these observations suggest distinct pathogenesis of the various LC phenotypes. We extended prior reports suggesting that recent EBV reactivation is associated with LC, by demonstrating that these associations primarily involve fatigue and neurologic LC symptoms. We also made the potentially novel observation that CMV seropositivity has an unexpected, negative association with LC, which, in turn, is masked to some degree by HIV infection and serological evidence suggesting EBV reactivation. Nevertheless, the presence of LC symptoms could not be completely explained by the viral coinfections assessed in our study, suggesting that other factors must be important mediators of LC. In particular, it remains to be seen whether SARS-CoV-2 persistence in tissues may also play a role in LC, as suggested by recent uncontrolled case series of SARS-CoV-2–directed antiviral therapies (56–58). Ultimately, further investigation of SARS-CoV-2 and other viruses during both acute infection and convalescence will be needed to clarify the mechanisms driving LC and suggest interventions that may reverse or ameliorate these processes.

## Methods

### Study participants.

All participants in the Long-Term Impact of Infection with Novel Coronavirus (LIINC; NCT04362150) cohort with biospecimens available outside the acute window of SARS-CoV-2 infection were studied; the cohort procedures have been described in detail previously (59). Briefly, any adult with a history of SARS-CoV-2 infection identified on nucleic acid amplification testing, regardless of the presence of acute or postacute symptoms, was eligible to enroll more than 14 days following symptom onset and followed approximately every 4 months thereafter. Participants were recruited through a combination of mailings to all individuals testing positive at two academic medical centers as well as clinician- and self-referrals, as described elsewhere (59). We also deliberately enriched the cohort for people with HIV by notifying all eligible individuals testing positive for COVID-19 at two university-affiliated HIV clinics, which allowed us to assess the association between HIV and LC symptoms.

Data regarding the acute period of COVID-19 (including number, type, and severity of symptoms; hospitalization; and COVID-19 treatment), as well as demographics and medical comorbidities, were collected by self-report at the first visit and verified through review of medical records whenever possible. At each visit, participants were queried regarding the presence of 32 symptoms derived from the US Centers for Disease Control COVID-19 symptom list (60) and the Patient Health Questionnaire somatic symptom scale (61). Importantly, participants were specifically asked to describe symptoms only if they were new or worse compared with the period prior to COVID-19 (preexisting symptoms were not considered to represent LC). Participants were also asked to assign themselves a score using a visual-analogue scale from 0 to 100 to indicate their overall health prior to COVID-19, at the worst point in their illness, and in the week prior to the visit.

### Biospecimen collection.

At each visit, whole blood was collected in EDTA tubes followed by density gradient separation and isolation of peripheral blood mononuclear cells and plasma as previously described (62). Serum was obtained concomitantly from serum-separation tubes for antibody testing. Both plasma and serum samples were stored at –80°F.

### EBV assays.

EBV antibody testing was performed on participant serum by ARUP laboratories. The EBV antibody panel included quantitative measures of anti-VCA IgG and IgM, anti-Nuclear Antigen (EBNA) IgG, and EA-D IgG. Results were considered positive in this analysis if units (U) per mL were within or higher than the indeterminate range of the assay (VCA IgG ≥18 U/mL; VCA IgM ≥36 U/mL; EBNA IgG ≥18 U/mL; early D Ag ≥9 U/mL). The VCA IgG, EBNA IgG, and EA-D IgG assays had upper limits of quantitation (>750 U/mL, >600 U/mL, and >150 U/mL, respectively). Quantitative EBV PCR testing was performed on a random subset of 50 study participants stratified by EA-D IgG positivity by ARUP laboratories (quantitative range, 2.6–7.6 log copies/mL). This assay also identifies detectable EBV DNA above and below the limit of quantitation.

### CMV assays.

CMV serostatus was assessed in duplicate on cryopreserved serum by qualitative ELISA (CMV IgG ELISA [GWB-BQK12C], Genway Biotech), with antibody index values of less than 0.9 considered negative, more than 1.1 considered positive, and between 0.9 and 1.1 considered indeterminate per manufacturer specifications. Levels greater than 0.9 were considered detectable in this study. For participants without available serum at study entry, subsequent visits up to 20 months following COVID-19 diagnosis were used for serostatus ascertainment, as while the prevalence of CMV is high in the general population, the incidence among seronegative adults is typically less than 1% per year (63).

### Biomarker and SARS-CoV-2 IgG analyses.

A subset of participants (*n* = 143) had circulating biomarker data available from previous testing (3, 39). Briefly, the fully automated HD-X Simoa platform (Quanterix) was used to measure biomarkers in blood plasma, including MCP-1, Cytokine 3-PlexA (IL-6, IL-10, TNF-α), IFN-γ–induced protein 10 (IP-10), IFN-γ, NFL, GFAP, and SARS-CoV-2 receptor-binding domain IgG according to the manufacturer’s instructions (Quanterix). Assay performance was consistent with the manufacturer’s specifications.

### Statistics.

Descriptive statistics were used to characterize the cohort, including median and 25% and 75% quartiles for continuous variables. In univariate analyses of binary variables, we performed 2-sided χ^2^ testing or Fisher’s exact testing (if any expected cell value was less than 5) for cross-tabular data and 2-sided Mann-Whitney *U* or Kruskal-Wallis tests (for multiple comparisons with Dunn correction) to compare variables across LC groups, symptom groups, and EBV antibody results. Covariate-adjusted binary logistic regression models were performed to determine independent associations between variables and LC/symptom/antibody results. Continuous biomarker data used in binary regression models were log_10_ transformed to achieve normality and divided by the IQR for each individual biomarker in order normalize the effect size across variables. All *P* values are 2 sided. Prism version 9.1.2 (GraphPad Software) and SPSS version 28.0.1.1 (IBM) software were used for analyses. *P* values of less than 0.05 were considered significant.

### Study approval.

All participants provided written informed consent. The study was approved by the Institutional Review Board at the UCSF.

## Author contributions

MJP, SGD, PWH, and TJH designed the study, which was supported through funding to MAS, JDK, JNM, SGD, PWH, and TJH. MJP, RH, JDK, JNM, SGD, and TJH designed the cohort. DR, SEM, RH, VT, LT, MD, LHN, MB, and AR collected clinical data and biospecimens, which were processed by TMD, DR, SEM, LT, NSI, and AB in the laboratory of TJH. Specimens were analyzed by TMD, DR, SEM, GBE, FC, BCY, AC, JWW, and CJP. SL, SAG, and JYC processed and managed data. MJP and TJH performed and/or interpreted the statistical analyses. MJP, SGD, PWH, and TJH drafted the initial manuscript, with input from AND, JH, MAS, MSD, PYH, JDK, and JNM. All authors edited, reviewed, and approved the final manuscript.

## Supplementary Material

Supplemental data

ICMJE disclosure forms

## Figures and Tables

**Table 1:**
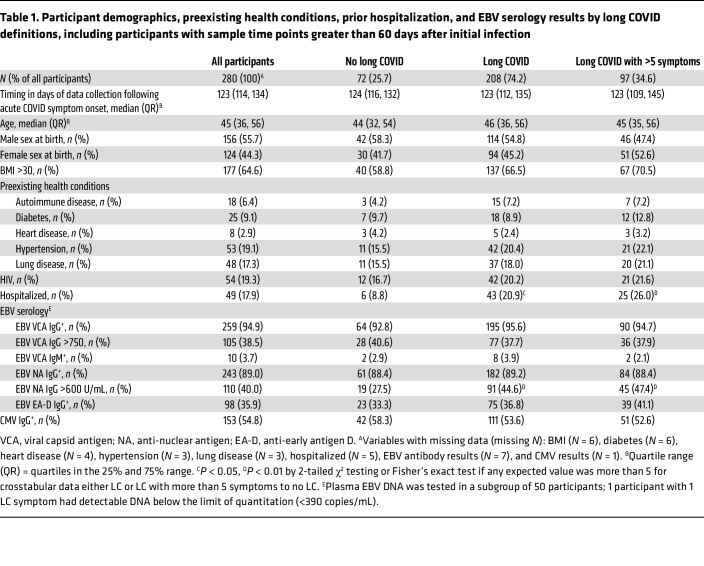
Participant demographics, preexisting health conditions, prior hospitalization, and EBV serology results by long COVID definitions, including participants with sample time points greater than 60 days after initial infection

## Version 1 12/01/2022

In-Press Preview

## Version 2 02/01/2023

Electronic publication
