# Supplementary material for: Chronic viral coinfections differentially affect the likelihood of developing long COVID
Source: J Clin Invest. 2023 Feb 1;133(3):e163669. doi: 10.1172/JCI163669 (PMC9888380; doi:10.1172/JCI163669)
Supplement: Supplemental data [file jci-133-163669-s021.pdf]

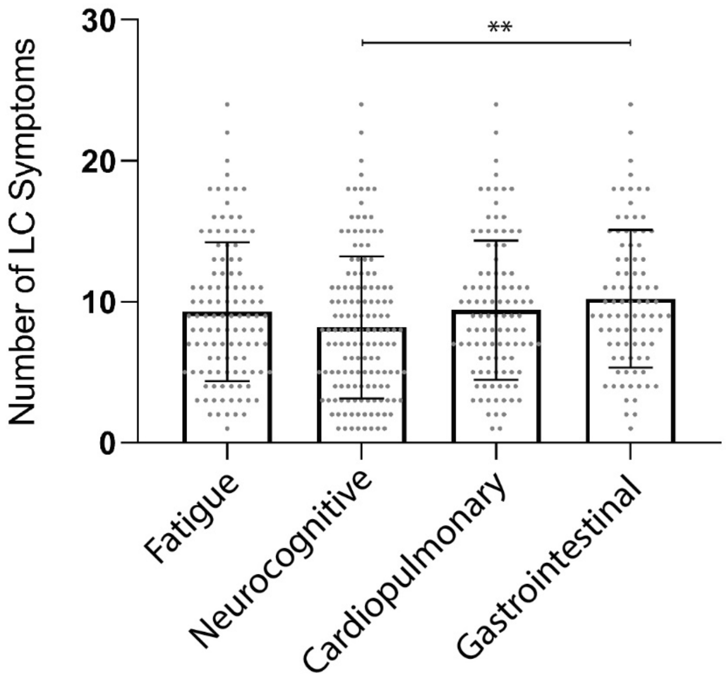

Supplemental Figure 1. Number of Long COVD (LC) symptoms at the time of sample collection (median 4 months following SARS-CoV-2 PCR diagnosis) by LC symptom phenotype. Bars and lines represent mean and standard deviation. ** P <0.01 as determine by Kruskal-Wallis test with Dunn correction for multiple comparisons. Each point represents a study participant.

Supplemental Table 1. Subgroup analysis of plasma EBV DNA testing and other factors in subgroups of randomly selected participants stratified by Early Antigen-Diffuse (EA-D) IgG positivity.

	EA-D IgG Negative	EA-D IgG Positive
N	25	25
Plasma EBV DNA Positive [n (%)]	1 (4)[a]	0 (0)
Timing in days of data collection following acute COVID symptom onset [median (QR)][b]	126 (120, 144)	121 (101, 128)
Age [Median (QR)]	42 (32, 55)	49 (37, 61)
Male Sex	18 (72)	12 (48)
BMI >30	15 (60)	17 (68)
Pre-existing Health Conditions		
HIV	3 (12)	5 (20)
Autoimmune Disease	0 (0)	3 (12)
Diabetes	1 (4)	2 (8)
Heart Disease	0 (0)	1 (4)
Hypertension	1 (4)	6 (24)
Lung Disease	5 (20)	7 (28)
Hospitalized	3 (12)	4 (16)
LC All	18 (72)	18 (72)
LC >5 Symptoms	9 (36)	9 (36)
Fatigue	10 (40)	13 (52)
Cardiopulmonary Symptoms	9 (36)	10 (40)
Gastrointestinal Symptoms	10 (40)	9 (36)
Neurocognitive Symptoms	13 (52)	15 (60)

BMI = body mass index; EBV = Epstein Barr Virus; Ag = antigen; LC = post-acute sequalae of SARS-CoV-2 infection

[a] One participant with one Long COVID symptom had detectable DNA below the limit of quantitation (<390 copies/mL)

[b] QR = 25%, 75% quartiles)

Supplementary Table 2. Relationship between HIV and EBV and CMV serological results in participants included in logistic regression models.

	HIV- (N=221)	HIV+ (N=52)	All Participants (N=273)
EBV EA-D IgG+	71 (32.1%)	27 (51.9%)**	98 (35.9%)
EBNA IgG>600 IU	87 (39.4%)	23 (44.2%)	110 (40.3%)
CMV IgG+	98 (44.5%)[a]	51 (98.1%)**	149 (54.8%)

[a] N=220 (1 HIV- participant did not have CMV IgG results)

** P <0.01 by two-tailed Chi Square Testing

Supplemental Table 3. Binary logistic regression results of circulating markers of inflammation by Long COVID symptom clusters with and without adjusting for CMV IgG results.

Variables in Regression	**Long COVID** OR (CI)[a]	**Long COVID >5 Sx** OR (CI)	**Fatigue** OR (CI)	**Neurocognitive Sx** OR (CI)	**Cardiopulmonary Sx** OR (CI)	**Gastrointestinal Sx** OR (CI)
NF-L	1.39 (0.79-2.45)	1.09 (0.54-2.22)	1.19 (0.71-2.01)	1.33 (0.8-2.2)	1.11 (0.66-1.86)	0.74 (0.42-1.31)
NF-L + CMV IgG	1.4 (0.78-2.52)	1.08 (0.53-2.24)	1.13 (0.66-1.94)	1.36 (0.81-2.29)	1.09 (0.64-1.87)	0.62 (0.37-1.21)
GFAP	0.94 (0.58-1.53)[b]	0.87 (0.49-1.52)	0.987 (0.61-1.56)	1.27 (0.81-1.98)	0.88 (0.55-1.41)	1.0 (0.61-1.64)
GFAP + CMV IgG	1.02 (0.49-2.12)[c]	0.87 (0.49-1.52)	1.0 (0.62-1.59)	1.24 (0.79-1.95)	0.86 (0.53-1.38)	1.02 (0.62-1.68)
IL-6	**2.09 (1.17-3.74)***	**3.19 (1.49-6.83)***	1.51 (0.94-2.43)	**1.9 (1.16-3.1)***	1.31 (0.82-2.08)	**2.11 (1.25-3.56)***
IL-6 + CMV IgG	**2.23 (1.21-4.1)***	**3.33 (1.51-7.32)***	1.44 (0.89-2.34)	**2.05 (1.22-3.45)***	1.35 (0.83-2.18)	**2.03 (1.19-3.46)***
TNFα	**1.69 (1.04-2.76)***	**2.33 (1.19-4.58)***	1.4 (0.9-2.18)	**1.86 (1.19-2.91)***	1.31 (0.85-2.03)	**1.67 (1.04-2.68)***
TNFα + CMV IgG	**1.77 (1.06-2.95)***	**2.4 (1.19-4.84)***	1.33 (0.84-2.1)	**2.04 (1.17-3.27)***	1.37 (0.86-2.16)	1.59 (0.98-2.59)
IFNγ	1.01 (0.66-1.55)	0.88 (0.51-1.54)	**0.63 (0.4-0.98)***	**0.66 (0.43-0.99)***	1.18 (0.79-1.77)	0.85 (0.55-1.31)
IFNγ + CMV IgG	1.02 (0.66-1.55)	0.88 (0.51-1.55)	**0.61 (0.39-0.96)***	**0.66 (0.43-0.995)***	1.18 (0.79-1.77)	0.83 (0.53-1.29)
IL-10	1.33 (0.84-2.1)	1.36 (0.74-2.49)	0.77 (0.51-1.17)	1.06 (0.77-1.48)	1.39 (0.96-2.01)	0.98 (0.68-1.41)
IL-10 + CMV IgG	1.33 (0.84-2.12)	1.35 (0.73-2.49)	0.73-0.47-1.14)	1.07 (0.77-1.49)	1.4 (0.96-2.03)	0.95 (0.65-1.4)
IP-10	1.4 (0.87-2.25)	1.18 (0.67-2.05)	0.88 (0.57-1.35)	1.12 (0.74-1.68)	1.12 (0.74-1.72)	0.84 (0.53-1.32)
IP-10 + CMV IgG	1.42 (0.86-2.34)	1.17 (0.65-2.11)	0.81 (0.51-1.28)	1.13 (0.74-1.73)	1.11 (0.71-1.73)	0.75 (0.46-1.23)
MCP-1	1.04 (0.66-1.64)	1.19 (0.65-2.16)	0.99 (0.64-1.54)	1.09 (0.72-1.65)	1.15 (0.75-1.79)	1.46 (0.9-2.36)
MCP-1 + CMV IgG	1.05 (0.66-1.67)	1.18 (0.65-2.16)	0.96 (0.62-1.5)	1.12 (0.74-1.69)	1.19 (0.76-1.85)	1.42 (0.88-2.3)

[a] Odds Ratio (OR) from [Log_{10}(biomarker)/ICR] = OR per IQR

[b] OR and 95% confidence intervals (CI) from binary logistic regression of biomarker variable alone (constant included in the model)

[c] OR and 95% CI from binary logistic regression of biomarker covariate adjusted for CMV IgG serostatus (constant included in the model)

IQR = interquartile range; Sx = symptoms; * $P<0.05$ in binary logistic regression models
